# Bone Mineral Density and Current Bone Health Screening Practices in Friedreich’s Ataxia

**DOI:** 10.3389/fnins.2022.818750

**Published:** 2022-03-14

**Authors:** Julia Dunn, Jaclyn Tamaroff, Anna DeDio, Sara Nguyen, Kristin Wade, Nicolette Cilenti, David R. Weber, David R. Lynch, Shana E. McCormack

**Affiliations:** ^1^Division of Endocrinology and Diabetes, Children’s Hospital of Philadelphia, Philadelphia, PA, United States; ^2^Ian M. Burr Division of Pediatric Endocrinology and Diabetes, Department of Pediatrics, Vanderbilt University Medical Center, Nashville, TN, United States; ^3^Department of Pediatrics, Perelman School of Medicine, University of Pennsylvania, Philadelphia, PA, United States; ^4^Division of Neurology, Children’s Hospital of Philadelphia, Philadelphia, PA, United States; ^5^Department of Neurology, Perelman School of Medicine, University of Pennsylvania, Philadelphia, PA, United States

**Keywords:** Friedreich Ataxia (FRDA), bone mineral density–BMD, fractures–bone, bone health, mitochondria

## Abstract

**Introduction:**

Friedreich’s Ataxia (FRDA) is a progressive neurological disorder caused by mutations in both alleles of the *frataxin (FXN)* gene. Impaired bone health is a complication of other disorders affecting mobility, but there is little information regarding bone health in FRDA.

**Methods:**

Dual energy X-ray absorptiometry (DXA) scan-based assessments of areal bone mineral density (aBMD) in individuals with FRDA were abstracted from four studies at the Children’s Hospital of Philadelphia (CHOP). Disease outcomes, including the modified FRDA Rating Scale (mFARS), were abstracted from the FRDA Clinical Outcomes Measures Study (FACOMS), a longitudinal natural history study. A survey regarding bone health and fractures was sent to individuals in FACOMS-CHOP.

**Results:**

Adults with FRDA (*n* = 24) have lower mean whole body (WB) (–0.45 vs. 0.33, *p* = 0.008) and femoral neck (FN) (–0.71 vs. 0.004, *p* = 0.02) aBMD Z-scores than healthy controls (*n* = 24). Children with FRDA (*n* = 10) have a lower WB-less-head (–2.2 vs. 0.19, *p* < 0.0001) and FN (–1.1 vs. 0.04, *p* = 0.01) aBMD than a reference population (*n* = 30). In adults, lower FN aBMD correlated with functional disease severity, as reflected by mFARS (*R* = –0.56, *p* = 0.04). Of 137 survey respondents (median age 27 y, 50% female), 70 (51%) reported using wheelchairs as their primary ambulatory device: of these, 20 (29%) reported a history of potentially pathologic fracture and 11 (16%) had undergone DXA scans.

**Conclusions:**

Low aBMD is prevalent in FRDA, but few of even the highest risk individuals are undergoing screening. Our findings highlight potential missed opportunities for the screening and treatment of low aBMD in FRDA.

## Introduction

Friedreich’s Ataxia (FRDA) is a progressive neurological disorder that affects about 1 in 50,000 individuals. FRDA is caused by the presence of a GAA triplet expansion on both alleles of the *FXN* gene in most (about 96%) of cases, and by a GAA triplet expansion on one allele and a point mutation or deletion on the other in a minority (about 4%) of cases (Cossée et al., 1999). The *FXN* gene encodes frataxin, a protein that facilitates assembly of iron-sulfur clusters within the body (Fox et al., 2019). The length of the shorter of the two triplet expansions on the *FXN* alleles correlates with residual frataxin production and disease severity (Filla et al., 1996).

The primary symptom of FRDA is ataxia, and therefore there are a spectra of mobility concerns in affected individuals. Impaired mobility could adversely impact bone health in individuals with FRDA, although the complex mechanistic processes linking activity and bone health are not entirely understood, either in healthy populations or FRDA (Hind and Burrows, 2007). To our knowledge, prior studies of bone health in individuals with FRDA are limited to a single pilot study reporting deficits in adults compared to reference cohorts (Eigentler et al., 2014). On the basis of impaired mobility in FRDA, the previous pilot study reporting low BMD in adults with FRDA, and bone health deficits in related disorders (Hind and Burrows, 2007; Gandhi et al., 2017; Zerbini et al., 2017; Ward et al., 2018), the objective of this study was to examine bone density in children and adults with FRDA, and investigate the current practices of screening and treatment of low aBMD in affected individuals.

## Materials and Methods

### Participants

Data for adults with FRDA were abstracted from three IRB-approved studies at CHOP and the University of Pennsylvania (Penn). In one study, healthy adult controls matched for age, sex, race, and BMI to individuals with FRDA were included. Pediatric data were abstracted from an additional ongoing fourth IRB-approved study. Reference pediatric data were abstracted from the Bone Mineral Density in Childhood Study (BMDCS) with appropriate data use agreements in place. Three healthy participants from BMDCS reference cohort were matched for age (±6 weeks), sex, and race to each child with FRDA (Kalkwarf et al., 2007). All studies collected demographic and anthropometric data, and participants completed dual-energy X-ray absorptiometry (DXA) scans. In total, 24 adults with FRDA, 24 adults without FRDA, 10 children with FRDA, and 30 healthy pediatric participants from BMDCS were included in the analysis (Supplementary Figure 1). DXA based assessments of lean mass and fat mass have been previously summarized (manuscript in revisions), but this is the first analysis related to bone. As spinal rods impact the results of some DXA outcomes, the whole body (WB) measurement for the single participant with spinal rods was excluded (*n* = 1), but femoral neck (FN) outcomes were included (Hsiao et al., 2019). FRDA-related outcomes for individuals with FRDA were abstracted from the Friedreich Ataxia Clinical Outcomes Measures Study (FACOMS), a multi-site, longitudinal, prospective natural history study with annual visits (Regner et al., 2012). To better understand fracture history and current bone health screening and management practices in individuals with FRDA, a survey was sent to individuals who were enrolled in FACOMS at CHOP. To test the extent to which survey respondents were representative of the larger population of individuals with FRDA, respondent characteristics were compared to those of the entire cohort of 449 individuals enrolled in FACOMS at CHOP.

### Dual Energy X-Ray Absorptiometry-Based Outcomes

All DXA scans were performed on a Hologic Horizon A densitometer (Hologic Inc., Marlborough, MA, United States) at CHOP between the years of 2015 to 2021. Areal bone mineral density (aBMD) was calculated (Truscott et al., 1996). In the adult cohort, DXA scans in all three studies measured the patient’s WB aBMD (*n* = 24 with FRDA, *n* = 24 without FRDA), and two studies additionally reported aBMD in the FN (*n* = 16 with FRDA, *n* = 24 without FRDA). aBMD outcomes were converted into Z-scores for age, race, and sex and T-scores relative to healthy white individuals aged 20–29 years of the same sex (Dimai, 2017; Shuhart et al., 2019). T-scores are used clinically to evaluate bone health in post-menopausal women and men age 50 y and older; however, we report all participants’ Z-scores in this analysis for consistency, including the small number over age 50 y. In the pediatric population, whole body less head (WBLH), rather than WB, aBMD was measured (Zemel et al., 2011). Pediatric aBMD Z-scores were calculated using reference data from BMDCS and adjusted for height (Zemel et al., 2011; Kindler et al., 2019, 2020). Z- and T-scores in adults were calculated using the standard clinical Hologic software. In total, 23 WB scans and 16 FN scans were available for adults with FRDA and 24 WB and FN scans were analyzed for adult controls. In children, 10 WBLH and FN scans were available for children with FRDA, and 30 WBLH and FN scans from healthy pediatric BMDCS participants were available (Supplementary Figure 2).

### Three-Day Diet Recall

Prior to the study visit, participants completed a 3-day diet record. The 3-day diet records were reviewed by the CHOP Center for Human Phenomic Science (CHPS) Bionutrition, and analysis of parameters of interest (intake of calcium, magnesium, phosphorus, vitamin D, vitamin K, and zinc) was performed using Nutrition Data System for Research software (v.2016, Minneapolis, MN).

### Plasma Calcium Concentrations

Plasma calcium measurements were obtained as part of screening for research study visits in one of the available studies and were measured using standard colorimetric techniques in the CHOP Hospital Laboratory. Vitamin D status and other bone turnover markers were not measured.

### Survey Data Collection

The survey on bone health in FRDA was sent to 473 unique email addresses associated with FACOMS participants followed at CHOP in August 2020 *via* Research Electronic data capture (REDCap) (Harris et al., 2009). The survey collected information on any assistive ambulatory devices used by the respondent; the number, sites, and mechanisms of fractures sustained; DXA scan history; risk factors for low BMD; use of supplements related to bone; and use of anti-osteoporotic medications (Supplementary Material; Kanis et al., 2013). For respondents who completed the survey more than once, only the most recent response was considered. Pathologic fractures were defined as occurring from trauma that would be otherwise unlikely to produce a fracture in the absence of impaired bone health (Adler, 1989). Any fractures reported to be sustained from low impact injuries (i.e., falls from standing height or below, as well as incidentally detected fractures) were considered potentially pathologic (Wren et al., 2012). Additionally, fractures of fingers or toes were excluded from analysis as has been done in other studies (Wasserman et al., 2018) because they are unlikely to be informative.

### Friedreich’s Ataxia-Related Outcomes

Disease-related outcomes from the closest visit within 6 months of either the participant’s DXA scan or survey response, as applicable, were abstracted from FACOMS. FRDA-related outcomes included disease duration, modified FRDA Rating Scale (mFARS) score, GAA repeat length on the shorter affected allele of the *FXN* gene, and age at FRDA symptom onset. All outcomes are potential indices of FRDA disease severity, though GAA length reflects genetic severity while mFARS score and disease duration reflect functional severity (Filla et al., 1996; Rummey et al., 2020).

### Statistical Analysis

All statistics were performed in Stata, version 16.1 (StataCorp LLC; College Station, TX, United States). Two sample *t*-tests or Wilcoxon rank sum tests were used to compare data between individuals with and without FRDA, based on normality. Spearman correlation analyses were used to evaluate the association between two outcomes of interest. The Shapiro-Wilk test was used to test for normality.

## Results

### Participant Characteristics

Twenty-four adults with FRDA with median age 26 years (IQR 23–45) and median BMI 23.1 kg/m^2^ (IQR 21.2–28.1) were compared to twenty-four healthy controls with similar overall distribution of age, weight, height, and BMI (Table 1). Additionally, 10 children with FRDA with median age 14 years (IQR 12–17) and mean BMI Z-score –0.13 (SD 2.04) were compared to a reference cohort (*n* = 30) matched for age, race, and sex (Table 2).

**TABLE 1:** Participant characteristics and bone mineral density data for adults with and without Friedreich’s Ataxia (FRDA).

	Controls (*n* = 24)	FRDA (*n* = 24)
Sex (%F, n)	50% (*n* = 12)	37.5% (*n* = 9)

	**Median (IQR)**	

Age (years)	29 (25, 43)	26 (23, 45)
*Characteristics of FRDA (FRDA only)*		
GAA repeat length on the least affected allele (base pairs) (*n* = 22 bp, *n* = 2 pm)	–	533 (300, 715)
mFARS score (*n* = 22)	–	48 (40, 61)
Age of onset (years) (*n* = 24)	–	13 (10, 16)
Disease duration (years) (*n* = 22)	–	17 (13, 23)
*Anthropometrics*		
Weight (kg)	75.3 (63.9, 83.0)	67.3 (60.5, 80.4)
Height (cm)	170.4 (163.5, 177.4)	169.6 (166.3, 174.1)
BMI (kg/m^2^)	24.5 (22.1, 27.7)	23.1 (21.2, 28.1)
*Bone mineral density*		

	**Mean ± SD**	

**Whole body aBMD Z-score** (*n* = 23 with FRDA, *n* = 24 without)**	0.33 ± 0.85	–0.45 ± 1.05
**Femoral neck Z-score** (*n* = 24 without FRDA, *n* = 16 with FRDA)*	0.004 ± 0.98	–0.71 ± 0.73

*Demographic and anthropometric data, characteristics of FRDA (FRDA only), and skeletal site specific aBMD for healthy adult controls and adults with FRDA. Age and anthropometric data were collected at the time of the DXA scan. Skeletal site specific aBMD is expressed as a Z-score. Time varying FRDA characteristics (mFARS score and disease duration) were obtained at the closest FACOMS visit within 6 months from the DXA scan; the time varying characteristics of FRDA (mFARS scores, and disease duration) were not recorded for individuals without a recent FACOMS visit. Values in bold text indicate a statistically significant difference between individuals with and without FRDA (p < 0.05) by a two-sample t-test.*

*p < 0.05; **p < 0.01.

**TABLE 2:** Participant characteristics and bone mineral density data for children with and without Friedreich’s Ataxia (FRDA).

	Reference (*n* = 30)	FRDA (*n* = 10)
Sex (%F, n)	50% (*n* = 15)	50% (*n* = 5)

	**Median (IQR)**	

Age (years)	14 (12, 17)	14 (12, 17)
*Characteristics of FRDA (FRDA only)*		
GAA repeat length on the least affected allele (base pairs) (*n* = 9 bp, *n* = 1 pm)	–	867 (783, 900)
mFARS score (*n* = 10)	–	39 (32, 48)
Age of onset (years) (*n* = 10)	–	7 (5, 8)
Disease duration (years) (*n* = 10)	–	6 (4, 9)
*Anthropometrics*		
Weight (kg)	55.4 (41.7, 62)	53.8 (38, 72.9)
Height (cm)	159.4 (150.0, 168.1)	157.2 (145.4, 167.4)
Height Z score (mean ± STD)	0.24 ± 0.85	–0.16 ± 0.77
BMI (kg/m^2^)	20.3 (17.8, 22.7)	21.2 (16.6, 26)
BMI Z score (mean ± STD)	0.17 ± 0.93	–0.13 ± 2.04
*Bone mineral density*		

	**Mean ± SD**	

Whole body less head aBMD Z-score***	0.19 ± 0.79	–2.2 ± 1.7
Femoral neck Z-score*	0.04 ± 1.0	–1.1 ± 1.4

*Demographic and anthropometric data, characteristics of FRDA (FRDA only), and skeletal site specific aBMD for a healthy reference population of children and children with FRDA. Age and anthropometric data were collected at the time of the DXA scan. Skeletal site specific aBMD is expressed as a height-adjusted Z-score. Time varying FRDA characteristics (mFARS scores, and disease duration) were obtained at the closest FACOMS visit from the DXA scan. Values in bold text indicate a statistically significant difference between individuals with and without FRDA (p < 0.05) by a two-sample t-test.*

*p < 0.05; ***p < 0.001.

### Bone Density in Individuals With Friedreich’s Ataxia

**FIGURE 1:**
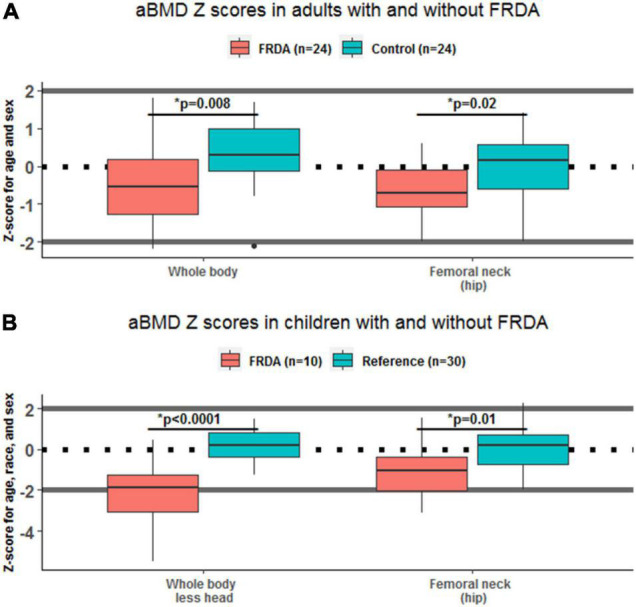
Areal bone mineral density (aBMD) Z-scores in adults and children, with and without Friedreich’s Ataxia (FRDA). Box plots show the difference between skeletal site specific aBMD Z-scores in individuals with FRDA (red) and controls (green), stratified by age. Statistically significant differences between FRDA and healthy controls are indicated above each pair of boxes. Panel **(A)** shows results in adults ages 18–55 y, and panel **(B)** shows results in children under 18 y.

Adults with FRDA had WB aBMD Z-scores that were lower than similarly aged adults without FRDA (difference of –0.78, 95% CI: –1.34 to –0.21; *p* = 0.008). The same was true for hip FN aBMD Z-scores (difference of –0.72, 95% CI: –1.30 to –0.13; *p* = 0.02) (Table 1 and Figure 1). Children with FRDA had a mean height Z-score of –0.16 ± 0.77, nominally but not statistically significantly lower than the mean reference cohort height Z-score score of 0.24 ± 0.85 (*p* = 0.20). Both the mean WBLH (difference of –2.36, 95% CI: –3.15 to –1.58, *p* < 0.0001) and FN (difference of –1.11, 95% CI: –1.93 to –0.28, *p* = 0.01) height-adjusted aBMD Z-scores of children with FRDA were significantly lower than the healthy age, sex, and race matched reference cohort (Table 2 and Figure 1, Zemel et al., 2011).

For this study, we reported Z-scores in adults 50 y and older so they could be analyzed with the balance of the cohort. However, for clinical evaluations, T-scores are reported in males over 50 years and post-menopausal females (Shuhart et al., 2019). In adults older than 50 years, WB T-scores of participants with (–0.86 ± 1.27, *n* = 5) and without (–0.52 ± 0.91, *n* = 5) FRDA were nominally lower but not statistically different in this small sample, as were the FN T scores of participants with (–2.8 ± 0.42, *n* = 2) and without (–1.7 ± 1.53, *n* = 5) FRDA.

### Nutrition in Friedreich’s Ataxia

In evaluating intake of bone-relevant nutrients (calcium, phosphorous, magnesium, zinc, vitamin K, and vitamin D), we found that few adults with FRDA were meeting the RDA for any of these with food alone (Institute of Medicine [US], 2011; National Academies of Sciences, Engineering, and Medicine, Health and Medicine Division, and Food and Nutrition Board, 2019). For example, only 21% of individuals met the RDA for calcium over the 3 days and none met the RDA for vitamin D. Only 3/19 (16%) took a multivitamin or vitamin D supplement and 2/19 (11%) took supplementary calcium. Supplementary Table 1 includes results for both individuals with FRDA and healthy controls. We did not detect a correlation between nutrient intake (calcium or vitamin D from diet and supplements) and FN or WB aBMD in adults with FRDA. Additionally, we examined blood calcium concentrations which were obtained in a subset of adults, and found that in both FRDA (*n* = 11) and healthy controls (*n* = 24) all participants were in the normal range (8.5–10.5 mg/dL).

### Relationship Between Bone Health and Features of Friedreich’s Ataxia

**FIGURE 2:**
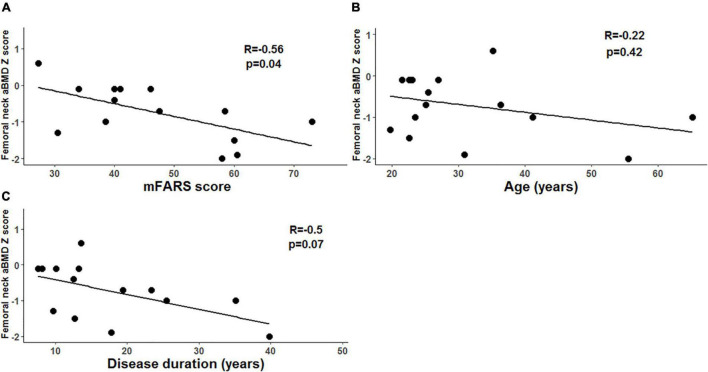
Femoral neck areal bone mineral density (aBMD) and markers of disease severity in adults with Friedreich’s ataxia (FRDA). **(A)** In adults with FRDA, mFARS scores (higher scores indicate more severe disease) are negatively correlated with femoral neck aBMD Z-scores. **(B)** We did not detect a correlation between disease duration and femoral neck aBMD Z scores. **(C)** We did not detect a correlation between age and femoral neck aBMD Z scores.

Correlations between skeletal site Z-scores and characteristics of FRDA (including GAA repeat length on the shorter allele, age of symptom onset, FRDA disease duration, and mFARS score) were tested. In adults with FRDA, there was a statistically significant negative correlation between hip FN Z-score and mFARS score (*R* = –0.56, *p* = 0.04), though not with age and not with disease duration (Figure 2). In children, we did not detect any significant correlations between either of the two skeletal site Z-scores and any of the characteristics of FRDA tested.

### Bone Health Survey

After disseminating the survey to 473 unique email addresses associated with individuals enrolled in FACOMS at CHOP and excluding duplicates (*n* = 17), we received 137 unique responses (*n* = 107 adults, *n* = 30 children, for a rate of 29% survey completion). The median age of survey respondents was 27 years (IQR 20–37) and there were 65 (47%) self-identified males, 68 (50%) self-identified females, and four individuals who identified as transgender, other, or unreported. There was no significant difference between the GAA repeat length (median 666 base pairs, IQR 499–790 bp) and age of symptom onset (median 11 years, IQR 7–15 years) of the survey respondents and the GAA repeat length (median 694 bp, IQR 521–800 bp) and age of symptom onset (mean 10 years, IQR 6–15 years) of the 449 individuals in FACOMS at CHOP, indicating similar disease severity between individuals who responded to the survey and the larger group to whom the survey was sent.

In terms of self-reported risk factors for low aBMD (Kanis et al., 2013), out of the 137 respondents, none reported consuming three or more alcoholic beverages per day, and five reported smoking tobacco products. Twenty-four individuals reported ever having taken steroid medications, six report current steroid use, and 11 reported having diabetes mellitus.

Twenty of the 70 respondents primarily using wheelchairs (29%) and 16 of the 67 respondents not primarily using wheelchairs (24%) reported having sustained a potentially pathologic fracture (defined as incidentally detected fractures, or fractures from falls at standing height or below), rates which were not statistically different. Despite the relatively high rate of potentially pathologic fractures, only 11 of the 70 wheelchair-users (16%) and 5 of the 67 respondents (7.5%) not using wheelchairs reported ever having had a DXA scan (Supplementary Figure 1), rates which were also not statistically different. Use of calcium (or increased calcium in the diet), vitamin D3, or a multivitamin for bone health protection was common, with 83/137 (60.5%) reporting use. On the other hand, use of pharmacologic intervention for low BMD was uncommon, with only five individuals reporting any such treatment, including four individuals on a bisphosphonate, and one on denosumab.

## Discussion

Our study had several key findings. First, in adults with FRDA, WB, and FN aBMD Z-scores were lower than in healthy controls (Table 1 and Figure 1). Also, lower bone density at the hip was correlated with mFARS score reflecting more substantial disease burden (Figure 2). Similar patterns were present in children with FRDA, who had lower WBLH and FN Z-scores than a healthy reference cohort (Table 2 and Figure 1). To our knowledge, there is only one prior study examining the bone health in individuals with FRDA (*N* = 28 adults) (Eigentler et al., 2014). Similar to the present study, the previous report found lower aBMD in FN relative to normative values. They also found a negative correlation between ataxia severity, GAA repeat expansion, and FN BMD in FRDA. In addition, they reported that 21% of adults with FRDA had at least one lifetime fracture (compared to our adult reported lifetime fracture rate of 40%), however, no information regarding the number, site, or mechanisms of fractures was available (Eigentler et al., 2014). Our study also examined additional skeletal sites (including WB), and added pediatric measurements. Together with the previous work, our study adds to the evidence that impaired bone health is prevalent in FRDA.

In considering potential explanations for the substantial prevalence of impaired BMD in FRDA, the correlation between disease severity (indicated by higher mFARS scores) and lower aBMD in adults with FRDA deserves attention. While our study was not designed to evaluate the direction of this effect, we speculate that severe FRDA may cause both the higher mFARS score and lower aBMD Z-score. Multiple factors may contribute. For example, individuals with more severe FRDA will have a worse mFARS score and may also be less likely to undertake weight-bearing physical activity, which would in turn lead to lower aBMD (Jiang et al., 2006; Ward et al., 2018). Indeed, in other disorders that adversely impact mobility (e.g., rheumatoid arthritis and Duchenne Muscular Dystrophy), poor bone health is a known complication and can lead to adverse clinical outcomes, such as pain and decreased ambulation (Zerbini et al., 2017; Ward et al., 2018). Alternatively, or in addition, severe FRDA may be associated with developmental and/or acquired deficits in skeletal muscle that also contribute to decreased aBMD. Previous analyses of this cohort have shown that individuals with FRDA have less appendicular lean mass (i.e., lean mass in arms and legs, reflecting mostly muscle) than individuals without FRDA (manuscript in revision).

Other factors including habits, medications, and comorbidities may also contribute in an additive way to low bone density in FRDA. In a previous study of genetic primary mitochondrial diseases, many individuals had more than one risk factor for low aBMD (Gandhi et al., 2017). Additional potential contributory risk factors, including smoking, drinking 3 or more alcoholic beverages per day, and steroid use were collected *via* survey in this study. While tobacco and excess alcohol use were not common, 24 of the 137 respondents (17.5%) reported having ever taken steroids and six (4.4%) reported ongoing steroid use. While systemic steroids are not considered standard of care in FRDA, they have been previously tested in at least one clinical trial (Patel et al., 2019), and the prevalence of current systemic steroids use in FACOMS has been previously reported as 3.5% (23/641), similar to our study (Shinnick et al., 2016). While individuals from the survey may not take steroids specifically for FRDA, by virtue of their shared genetic background, individuals with FRDA may also have excess rates of other conditions that require steroid treatment. For example, one study found that inflammatory bowel disease (IBD) was 3.5 times more common in a cohort of 641 individuals with FRDA than in the general population (Shinnick et al., 2016). IBD is associated with decreased bone health and increased risk for osteoporosis, in part related to associated steroid use (Chedid and Kane, 2020). Other concurrent inflammatory conditions could also contribute.

In terms of a nutritional component impacting bone health, we report that, at most half of adults with FRDA are meeting the recommended daily allowance (RDA) of bone-important nutrients including calcium, magnesium zinc, vitamin D, and vitamin K. Most met requirements for phosphorous (Supplementary Table 1). Rates of supplementation are low. Recognizing the limitations of brief, current, self-reported data, prospective, longitudinal studies including laboratory correlates of dietary intake are needed to determine the impact of these potential nutritional deficiencies on bone in individuals with FRDA. Diet and supplement use may change over time in ways that are not reflected with this 3-day instrument. Still, these findings highlight a potential opportunity to incorporate nutrition into future interventional studies to optimize bone health in individuals with FRDA. Additionally, we did not find any hypocalcemia in individuals with FRDA, though hypocalcemia would be expected to be a very late finding in nutritional calcium and/or vitamin D deficits. We did not find overt evidence of hypercalcemia, related, for example, to immobility and/or disorders of PTH homeostasis. In future studies, evaluating measures of bone turnover markers and calcium homeostasis may enhance our understanding.

Mechanistic processes directly linking FRDA and low aBMD remain largely unexplored. Frataxin deficiency in individuals with FRDA may lead to multiple impairments in cellular processes, but no studies have specifically investigated the effect of frataxin deficiency in bone cells (i.e., osteoblasts and osteoclasts). Additionally, studies have shown that impaired mitochondrial function can lead to poor bone health (Gandhi et al., 2017; Dobson et al., 2020), and frataxin deficiency is associated with decreased mitochondrial OXPHOS capacity, which may be relevant in mitochondria-rich osteoclasts. While it is known that individuals with FRDA can have decreased tissue-specific mitochondrial function in the setting of frataxin deficiency, to date no studies have investigated the link between mitochondrial function and bone specifically in FRDA (Papiha et al., 1998; Yamasaki and Hagiwara, 2009; Kim et al., 2012; Pandolfo and Hausmann, 2013). It may be relevant to examine whether the bone findings in humans with FRDA have correlates in preclinical models of the disease to help better understand the overall pathogenesis of FRDA-related bone disease.

Another potential mechanistic link between low aBMD and FRDA is impaired signaling by the transcription factor nuclear factor erythroid 2-related factor (Nrf2). Nrf2 is expressed in cells critical to bone turnover including osteoblasts, osteocytes, and osteoclasts. Nrf2 knock-out mice have lower BMD (Sun et al., 2015a,b). In FRDA there is decreased Nrf2 signaling in multiple tissues, and omaveloxolone, a Nrf2 activator, has recently been tested in clinical trials and demonstrated benefit for a summary measure of clinical disease severity in FRDA (La Rosa et al., 2021; Lynch et al., 2021). In the omaveloxolone clinical trial, over 48 weeks, one fracture occurred in the placebo group and none in the treatment group, an insignificant difference (Lynch et al., 2021). If omaveloxolone is eventually used more widely, future studies could consider assessing possible direct and/or indirect effects of treatment on aBMD. Since Nrf2 is pleotropic, changes in aBMD could be from an anabolic effect on bone, or could be indirect, for example, if individuals are able to perform more weight-bearing physical activity while on treatment.

Results from our survey showed that rates of potentially pathologic fractures, use of DXA scans to evaluate BMD, and use of anti-osteoporotic medications were similar between individuals primarily using wheelchairs and those not primarily using wheelchairs. Despite the substantial rate of potentially pathologic fractures, very few individuals have undergone DXA screening. In addition, low aBMD is one possible contributor to fracture risk in FRDA, but it is certainly not the only contributor. For example, diabetes can be a comorbidity of FRDA, and diabetes in individuals without FRDA has been shown to increase lifetime risk of fracture (Weber et al., 2015). Also, another contributor to fracture risk is exposure to trauma. While some individuals with FRDA remain active and may experience falls, others may participate less in activities that may lead to fracture (e.g., recreational sports). The mechanisms of fracture in chronic disorders are complex because of these numerous interacting factors. Nonetheless, given the difference in aBMD between FRDA and control cohorts, we suspect that low aBMD is one factor that could contribute to excess rates of pathologic fractures.

There was a variety of types of bones broken based on survey response (Supplementary Figure 1). There was no difference between the types of bones fractured between those in wheelchairs vs. not in wheelchairs. We note that arm and ankle fractures were common, similar to a study in pediatrics that reported forearm fractures as most common (Naranje et al., 2016). Types of bones fractured in this population may be different than in epidemiologic studies in young adults as mechanisms are likely different. Studies in young adults suggest that fractures are often related to severe trauma (fall from above standing height, motor vehicle accident, and other high impact injuries) (Farr et al., 2017). While we did not find as many severe traumas in this cohort, prospective data collection would better answer this question. Additionally, epidemiologic patterns in young adults indicate increased fracture risk in males compared to females, likely due to exposures (Farr et al., 2017). We did not detect an increased risk of fractures in self-identified males with FRDA, and in fact found that 49% of females (33/68) and 32% of males (21/65) reported a fracture, though this did not meet statistical significance (*p* = 0.06).

Our study has several strengths and limitations. One strength is the inclusion of contemporaneously collected matched control cohort for the adults, and the carefully matched pediatric control BMDCS participant data. Another strength is that all DXA scans were performed on the same scanner and software for individuals with FRDA, thus obviating concerns regarding inter-scanner variability. Our survey also included a relatively large number of individuals, considering the rarity of the disease, and our survey respondents were overall very similar to a very large natural history cohort of individuals with FRDA, enhancing generalizability.

Limitations of our study include the relatively small sample size of the cohort undergoing DXA scans, particularly in the pediatric group (*n* = 10). However, our findings in this relatively rare disorder are important to both build on previous work and motivate future studies that include prospective data collection. Another potential limitation is that our analysis of the pediatric population relied on population reference data instead of contemporaneous controls, though the Z-scores do take into account age, race, gender, and height, and are the usual way of evaluating pediatric bone health in clinical practice. Finally, our survey data relied on participant report, and mechanisms of fracture can be difficult to elucidate accurately *via* survey. Future prospective studies could enrich our understanding by obtaining detailed supporting medical information for incident fractures.

In summary, in this study, as compared to healthy reference cohorts, children and adults with FRDA had lower aBMD, both overall (i.e., WB) and at the clinically relevant FN skeletal site. Also, low bone density was correlated with clinically worse disease in adults. For adults with low bone density, there are approved medications that in other populations have been shown to meaningfully reduce the risk of future fracture. These medications are also used in children with low bone density, in particular those who fracture related to fragile bones (Zacharin et al., 2021). One study in different condition characterized by early onset of mobility impairment, Duchenne Muscular Dystrophy, found that boys (ages 6–16 years) treated with bisphosphonates had increased aBMD and potentially decreased risk of incident fracture (Zacharin et al., 2021). In FRDA, despite the apparent prevalence of low bone density and the capacity for screening to lead to actionable information, our survey indicated that very few individuals have undergone a screening DXA scan, even those who use wheelchairs who are likely at greatest risk for low bone density. To address this gap in screening, updated consensus guidelines on the utilization of screening DXA scans, as well as the use of calcium, vitamin D3, and bisphosphonates to treat low aBMD in FRDA are currently in development. Future studies could also test the effect of medications to impact bone density and fracture risk specifically in FRDA. Despite limited information on treatment of impaired bone health in FRDA, our findings suggest potential missed opportunities for the screening, prevention, and treatment of low aBMD that could improve wellbeing and extend function. The role of specific screening and intervention strategies to promote bone health in FRDA should be the focus of future investigation.

## Data Availability Statement

The raw data supporting the conclusions of this article will be made available by the authors, without undue reservation.

## Ethics Statement

The studies involving human participants were reviewed and approved by the Children’s Hospital of Philadelphia Institutional Review Board and/or the University of Pennsylvania Institutional Review Board. Written informed consent to participate in this study was provided by the participant or the participants’ legal guardian/next of kin.

## Author Contributions

JT and SM designed the study and analysis plan. SM and DL planned the initial studies. JD wrote the manuscript. JT, SM, DL, and DW critically reviewed the manuscript. AD, KW, and SN performed the study procedures. All authors provided feedback and helped shape the research, analysis, and manuscript.

## Author Disclaimer

The content is solely the responsibility of the authors and does not necessarily represent the official views of the National Institutes of Health.

## Conflict of Interest

The authors declare that the research was conducted in the absence of any commercial or financial relationships that could be construed as a potential conflict of interest.

## Publisher’s Note

All claims expressed in this article are solely those of the authors and do not necessarily represent those of their affiliated organizations, or those of the publisher, the editors and the reviewers. Any product that may be evaluated in this article, or claim that may be made by its manufacturer, is not guaranteed or endorsed by the publisher.
