# Supplementary material for: Bone Mineral Density and Current Bone Health Screening Practices in Friedreich’s Ataxia
Source: Front Neurosci. 2022 Mar 14;16:818750. doi: 10.3389/fnins.2022.818750 (PMC8964400; doi:10.3389/fnins.2022.818750)
Supplement: Supplementary file 1 [file Data_Sheet_1.docx]

**Supplementary Figure 1.** Fracture mechanisms and sites, and DXA scans in individuals with FRDA, stratified by wheelchair use.


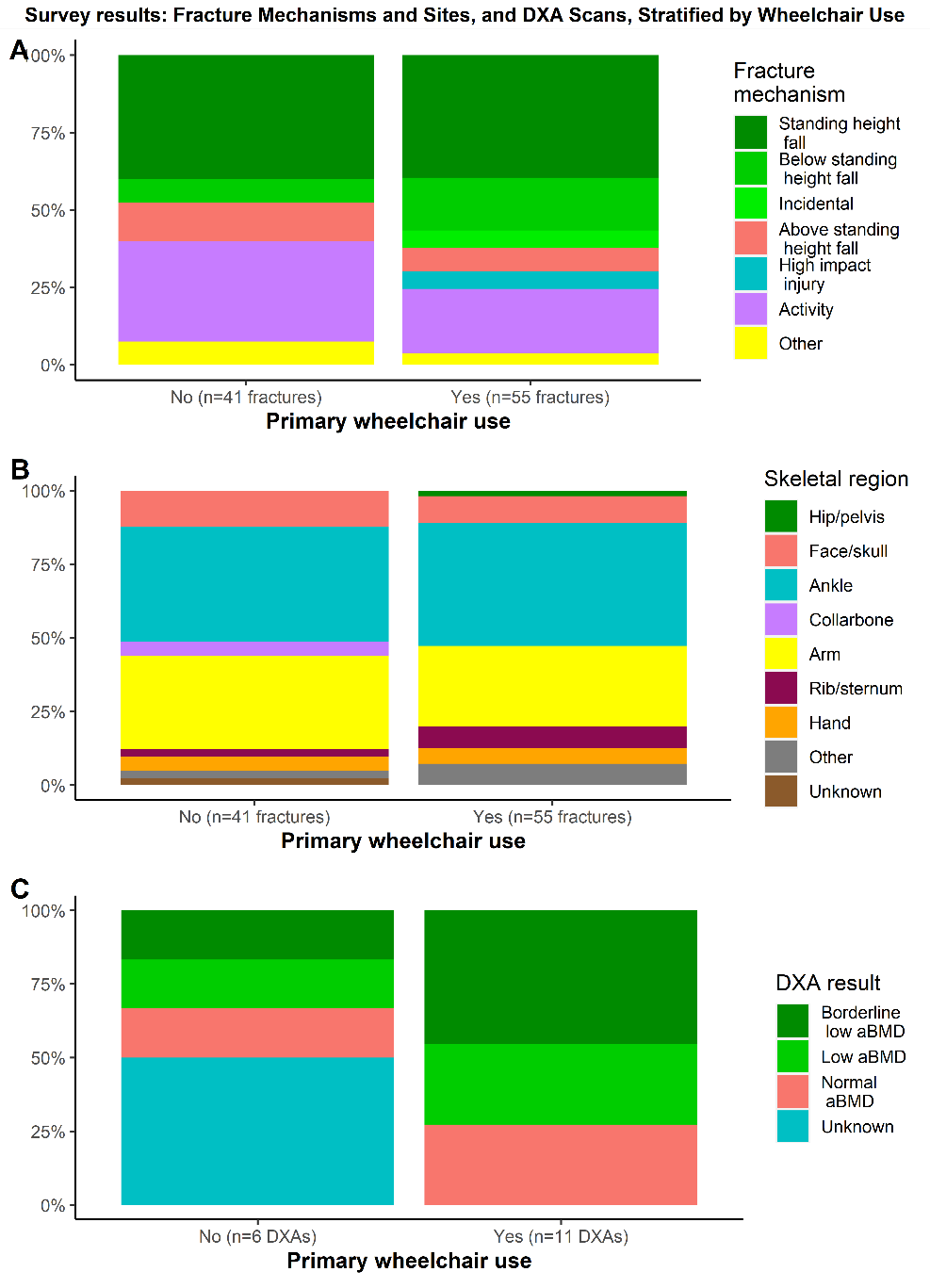


**Supplementary Figure 1. (A)** Fracture mechanisms are shown in individuals either using or not using wheelchairs as their primary device. Fractures of fingers and toes were excluded. Green indicated potentially pathologic fractures based on mechanisms (fall from standing height, below standing height, and incidentally detected). There was no difference in the number of potentially pathologic fractures in those who do and do not use wheelchairs primarily. **(B)** Specific bones that were broken, excluding finger and toe fractures, are shown. There was no difference in types of bones broken in those who do and do not use wheelchairs primarily. **(C)** DXA scan results are shown in individuals using and not using wheelchairs. There was no difference between the number of individuals undergoing DXA scans in individuals using or not using wheelchairs.

**Supplementary Figure 2.** Exclusion tree for participant analysis.


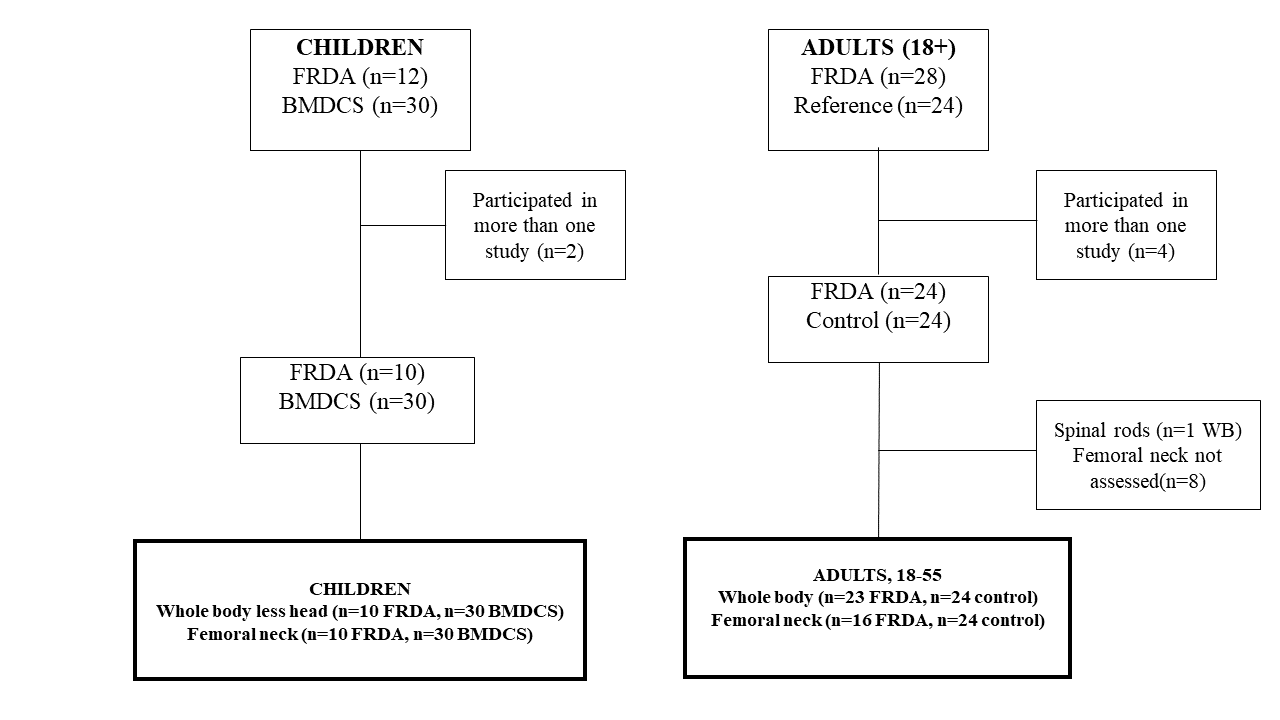


| **Nutrient (RDA)** | **Healthy controls (N=23):**  **% meeting RDA via diet**  **Median intake (IQR)** | **FRDA (N=19):**  **% meeting RDA via diet**  **Median intake (IQR)** |
| --- | --- | --- |
| Calcium (1000 mg) | 12/23 (52%)  1046 mg  (662, 1332) | 4/19 (21%)  706mg  (569, 894) |
| Phosphorous (700 mg) | 22/23 (96%)  1285mg  (1102, 1788) | 16/19 (84%)  1032mg  (815, 1272) |
| Magnesium |  |  |
| Females (310-320 mg) | 8/12 (67%)  338mg  (262, 375) | 0/6 (0%)  178mg  (169, 191) |
| Males (400-420 mg) | 2/11 (18%)  357mg  (253, 368) | 1/13 (8%)  225mg  (151, 234) |
| Vitamin D (15mcg) | 1/23 (4%)  5mcg  (3, 8) | 0/19 (0%)  3mcg  (1, 4) |
| Zinc | | |
| Females (8 mg) | 9/12 (75%)  11mg  (9, 14) | 3/6 (50%)  8mg  (7, 9) |
| Males (11 mg) | 9/11 (82%)  16mg  (13, 20) | 4/13 (31%)  10mg  (7, 12) |
| Vitamin K | | |
| Females (90 mcg) | 12/12 (100%)  185mcg  (142, 376) | 3/6 (50%)  94.10mcg  (70, 120) |
| Males (120 mcg) | 5/11 (45%)  105mcg  (62, 203) | 1/13 (8%)  54mcg  (41, 86) |

**Supplementary Table 1.** Dietary recall for bone related nutrition.

Self-reported 3-day diet records were summarized using automated software for vitamins and minerals relevant for bone health (these included food only, no supplements). We also queried self-reported medications and/or supplements. Of controls, 1/23 (4%) took a multi-vitamin, 2/23 (9%) took vitamin D, and 1/23 (4%) took calcium. Of individuals with FRDA, 2/19 (11%) took a multi-vitamin, 1/19 took vitamin D (5%), and 2/19 took calcium (11%).

**Supplementary Material: Survey on bone health in FRDA.** The survey on bone health in FRDA was sent to participants in FACOMS at CHOP.
