# Supplementary material for: Bone Mineral Density and Current Bone Health Screening Practices in Friedreich’s Ataxia
Source: Front Neurosci. 2022 Mar 14;16:818750. doi: 10.3389/fnins.2022.818750 (PMC8964400; doi:10.3389/fnins.2022.818750)
Supplement: Supplementary file 2 [file Data_Sheet_2.pdf]

Introduction and Fracture history

Please move from one survey to the next. If you do not have diabetes, there will be instructions when to stop. If you have diabetes, we ask that you complete the entirety of the survey.

Thank you for completing this survey!

Participant ID number ________________________

Name (First name Last name) ________________________

Date of Birth ________________________

What is your gender?
- ◯ Male
- ◯ Female
- ◯ Transgender
- ◯ Other
- ◯ Prefer not to say

Who is completing this survey?
- ☐ Self
- ☐ Self with caregiver
- ☐ Parent
- ☐ Spouse
- ☐ Other caregiver

What is your most recent weight?
(in pounds) ________________________

When was this weight checked?
- ◯ At a doctor's visit in the last year
- ◯ At a doctor's visit more than a year ago
- ◯ At home
- ◯ Other

What is your most recent height?
(in inches) ________________________

When was this height measured?
- ◯ At a doctor's visit in the last year
- ◯ At a doctor's visit more than a year ago
- ◯ At home
- ◯ Other

If you use a walking device, what do you use?

	Cane	2 cane/ crutches (Canadian Crutches)	Walker	Canine Assistance (Service Dog)	Wheelchair	Other	None
Primary Device	◯	◯	◯	◯	◯	◯	◯
Secondary device	◯	◯	◯	◯	◯	◯	◯

What age did you start using your primary walking device? ________________________

What age did you start using your secondary walking device?	______________________________
If you use an alternate walking device, what is it?	______________________________
Have you ever fractured (broken) a bone?	◯ Yes<br>◯ No
If yes, how many bones have you broken?	◯ 1<br>◯ 2<br>◯ 3<br>◯ 4<br>◯ 5<br>◯ more than 5
What is one bone that you have broken?<br>(There will be separate questions for each broken bone)	◯ Face/Skull<br>◯ Rib/Sternum<br>◯ Collarbone (Clavicle)<br>◯ Arm (including upper arm, elbow, lower arm, wrist)<br>◯ Hand<br>◯ Fingers or Toes<br>◯ Hip/Pelvis<br>◯ Thigh<br>◯ Lower leg, ankle, foot<br>◯ Spine (Vertebrae)<br>◯ Other<br>◯ Unknown
If you broke a different bone than listed, please list:	______________________________
How old were you when you broke this bone (years)?	______________________________
How did you break this bone?	◯ Fall (please choose this for any type of fall and there will be a question with additional details below)<br>◯ High impact injury (example: motor vehicle accident)<br>◯ During activity (examples: bicycling, skateboarding, playing a sport)<br>◯ Injury unknown (incidentally detected fracture)<br>◯ Other
If you broke it an alternate way, how did you break this bone?	______________________________
If you broke your bone from a fall, how high was it from?	◯ Above standing height (greater than 6 inches above standing)<br>◯ Standing height (within 5-6 inches from where head would be standing)<br>◯ Less than standing height (over 6 inches below where head would be if standing)

What is another bone that you have broken? (There will be separate questions for each broken bone)	◯ Face/Skull<br>◯ Rib/Sternum<br>◯ Collarbone (Clavicle)<br>◯ Arm (including upper arm, elbow, lower arm, wrist)<br>◯ Hand<br>◯ Fingers or Toes<br>◯ Hip/Pelvis<br>◯ Thigh<br>◯ Lower leg, ankle, foot<br>◯ Spine (Vertebrae)<br>◯ Other<br>◯ Unknown
If you broke a different bone than listed, please list:	__________________________________
How old were you when you broke this bone (years)?	__________________________________
How did you break this bone?	◯ Fall (please choose this for any type of fall and there will be a question with additional details below)<br>◯ High impact injury (example: motor vehicle accident)<br>◯ During activity (examples: bicycling, skateboarding, playing a sport)<br>◯ Injury unknown (incidentally detected fracture)<br>◯ Other
If you broke it an alternate way, how did you break this bone?	__________________________________
If you broke your bone from a fall, how high was it from?	◯ Above standing height (greater than 6 inches above standing)<br>◯ Standing height (within 5-6 inches from where head would be standing)<br>◯ Less than standing height (over 6 inches below where head would be if standing)
What is another bone that you have broken? (There will be separate questions for each broken bone)	◯ Face/Skull<br>◯ Rib/Sternum<br>◯ Collarbone (Clavicle)<br>◯ Arm (including upper arm, elbow, lower arm, wrist)<br>◯ Hand<br>◯ Fingers or Toes<br>◯ Hip/Pelvis<br>◯ Thigh<br>◯ Lower leg, ankle, foot<br>◯ Spine (Vertebrae)<br>◯ Other<br>◯ Unknown
If you broke a different bone than listed, please list:	__________________________________
How old were you when you broke this bone (years)?	__________________________________

How did you break this bone?	◯ Fall (please choose this for any type of fall and there will be a question with additional details below)<br>◯ High impact injury (example: motor vehicle accident)<br>◯ During activity (examples: bicycling, skateboarding, playing a sport)<br>◯ Injury unknown (incidentally detected fracture)<br>◯ Other
If you broke it an alternate way, how did you break this bone?	__________________________________
If you broke your bone from a fall, how high was it from?	◯ Above standing height (greater than 6 inches above standing)<br>◯ Standing height (within 5-6 inches from where head would be standing)<br>◯ Less than standing height (over 6 inches below where head would be if standing)
What is another bone that you have broken? (There will be separate questions for each broken bone)	◯ Face/Skull<br>◯ Rib/Sternum<br>◯ Collarbone (Clavicle)<br>◯ Arm (including upper arm, elbow, lower arm, wrist)<br>◯ Hand<br>◯ Fingers or Toes<br>◯ Hip/Pelvis<br>◯ Thigh<br>◯ Lower leg, ankle, foot<br>◯ Spine (Vertebrae)<br>◯ Other<br>◯ Unknown
If you broke a different bone than listed, please list:	__________________________________
How old were you when you broke this bone (years)?	__________________________________
How did you break this bone?	◯ Fall (please choose this for any type of fall and there will be a question with additional details below)<br>◯ High impact injury (example: motor vehicle accident)<br>◯ During activity (examples: bicycling, skateboarding, playing a sport)<br>◯ Injury unknown (incidentally detected fracture)<br>◯ Other
If you broke it an alternate way, how did you break this bone?	__________________________________
If you broke your bone from a fall, how high was it from?	◯ Above standing height (greater than 6 inches above standing)<br>◯ Standing height (within 5-6 inches from where head would be standing)<br>◯ Less than standing height (over 6 inches below where head would be if standing)

What is another bone that you have broken? (There will be separate questions for each broken bone)

- ◯ Face/Skull
- ◯ Rib/Sternum
- ◯ Collarbone (Clavicle)
- ◯ Arm (including upper arm, elbow, lower arm, wrist)
- ◯ Hand
- ◯ Fingers or Toes
- ◯ Hip/Pelvis
- ◯ Thigh
- ◯ Lower leg, ankle, foot
- ◯ Spine (Vertebrae)
- ◯ Other
- ◯ Unknown

If you broke a different bone than listed, please list:

____

How old were you when you broke this bone (years)?

____

How did you break this bone?

- ◯ Fall (please choose this for any type of fall and there will be a question with additional details below)
- ◯ High impact injury (example: motor vehicle accident)
- ◯ During activity (examples: bicycling, skateboarding, playing a sport)
- ◯ Injury unknown (incidentally detected fracture)
- ◯ Other

If you broke it an alternate way, how did you break this bone?

____

If you broke your bone from a fall, how high was it from?

- ◯ Above standing height (greater than 6 inches above standing)
- ◯ Standing height (within 5-6 inches from where head would be standing)
- ◯ Less than standing height (over 6 inches below where head would be if standing)

What is another bone that you have broken? Check all additional that apply

- ☐ Face/Skull
- ☐ Rib/Sternum
- ☐ Collarbone (Clavicle)
- ☐ Arm (including upper arm, elbow, lower arm, wrist)
- ☐ Hand
- ☐ Fingers or Toes
- ☐ Hip/Pelvis
- ☐ Thigh
- ☐ Lower leg, ankle, foot
- ☐ Spine (Vertebrae)
- ☐ Other
- ☐ Unknown

How old were you when you broke these bones? If multiple, please list- for example, Arm (10), spine (42)

____

How did you break these bones? If multiple, please list- for example, Arm (car accident), spine (fall)

____

If you broke your bone from a fall, how high was it from?	◯ Above standing height (greater than 6 inches above standing)<br>◯ Standing height (within 5-6 inches from where head would be standing)<br>◯ Less than standing height (over 6 inches below where head would be if standing)
Do you currently use glucocorticoids (steroid medication)?<br><br>Examples: hydrocortisone, prednisone, dexamethasone	◯ Yes, currently<br>◯ Yes, in the past but not currently<br>◯ Never
Do you currently smoke tobacco products?	◯ Yes<br>◯ No
Do you drink 3 or more alcoholic drinks per day?	◯ Yes<br>◯ No
Do you take any of the following to improve bone health? Check all that apply.	☐ Vitamin D supplementation<br>☐ Calcium supplementation<br>☐ Increased calcium in diet<br>☐ Multivitamin
Have you ever had a DXA scan to evaluate your bone density?	◯ Yes<br>◯ No
If you had a DXA for low bone density, what were the results?	◯ I was told I have low bone density<br>◯ I was told I have borderline low bone density<br>◯ I was told I have normal bone density<br>◯ I am not sure of the results
Have you ever been treated for low bone density?	◯ Yes<br>◯ No
If you have been treated for low bone density, what was the treatment?<br><br>Check all that apply.<br><br>Bisphphonates include: risendronate (Actonel, Atelvia), alendronate (Fosamax, Binosto), ibandronate (Boniva), zoledronic acid (Reclast, Zometa, Aclasta), pamidronate (Aredia), etidronate (Didronel)	☐ Calcium<br>☐ Vitamin D<br>☐ Bisphosphonates (names listed in question)<br>☐ Denosumab (Xgeva, Prolia)<br>☐ Teriparatide (Forteo) or abaloparatide (Tymlos)<br>☐ Romosozumab (Evenity)<br>☐ Selective estrogen receptor modulators: Rloxifene (Evista) or bazedoxifene (Conbriza, Viviant, Duavee, Duavive)<br>☐ Estrogen (oral contraceptives, estrogen patches)<br>☐ Calcitonin<br>☐ Other
If you had an alternate treatment for low bone mineral density, what was it?	______________________________
Which age range are you in?	◯ under 12 years old<br>◯ 12 to 18 years old<br>◯ over 18 years old

Please ensure this survey is filled out completely before clicking "submit".

Once you move to the next survey, you cannot return to this survey.

If you would like to return to this survey, please click "save and return".
